# Relationship between Sedentary Time, Physical Activity, and Health-Related Quality of Life in Spanish Children

**DOI:** 10.3390/ijerph18052702

**Published:** 2021-03-08

**Authors:** Manuel Ávila-García, María Esojo-Rivas, Emilio Villa-González, Pablo Tercedor, Francisco Javier Huertas-Delgado

**Affiliations:** 1 PA-HELP Physical Activity for Health Promotion Research Group, Department of Physical Education and Sport, Faculty of Sport Sciences, University of Granada, Camino de Alfacar 402, 18011 Granada, Spain; mavila@ugr.es (M.Á.-G.); maria.er46@gmail.com (M.E.-R.); tercedor@ugr.es (P.T.); 2 PROFITH Promoting Fitness and Health through Physical Activity Research Group, Department of Physical and Sports Education, Faculty of Education and Sport Sciences, Sport and Health University Research Institute (iMUDS), University of Granada, 52005 Melilla, Spain; 3 La Inmaculada Teacher Training Centre, PA-HELP Physical Activity for Health Promotion Research Group, University of Granada, Calle Joaquina Eguaras, 114, 18013 Granada, Spain; fjhuertas@ugr.es

**Keywords:** quality of life, physical activity, sedentary time, sex, school

## Abstract

Higher sedentary time and lower physical activity (PA) are associated with a poor health-related quality of life (HRQoL) in children. The aims of this study were: (1) to analyze the sedentary time, objectively measured PA levels (light, moderate, vigorous, and moderate-to-vigorous physical activity (MVPA)), and HRQoL dimensions (physical well-being, emotional well-being, self-esteem, family, friends, school, and total score) in children; and (2) to examine the association between sedentary time, PA levels, and HRQoL in children separately by sex. A total of 459 children (8.4 ± 0.4 years old, 50.54% males) from 15 schools in Granada (Spain) participated in the study. A tri-axial accelerometer was used to measure PA levels in the children for 7 consecutive days. The Revidierter KINDer Lebensqualitätsfragebogen (KINDL-R) questionnaire was used to determine the children’s HRQoL dimensions. The results showed that males presented more minutes engaged in MVPA than females. Both sedentary time and PA levels were associated with self-esteem and total score (all *p* < 0.05). In males, moderate and vigorous PA levels were associated with higher HRQoL, whereas light PA was associated with higher HRQoL in females. Future studies should take into account the use of activities with difference intensities in order to increase HRQoL in males and females.

## 1. Introduction

Today, a sedentary lifestyle is predominant across society, and the current situation with COVID-19 has intensified this situation [1]. The WHO recommends increasing children’s physical activity to 60 min per day in order to achieve health-related benefits [2]. Lower sedentary time and higher physical activity (PA) levels have been highlighted for their benefits to physical and psychosocial health in children [3], such as better physical fitness [4] and cognitive function [5]. Nevertheless, the majority of children and adolescents worldwide did not meet the physical activity guidelines [6]. In addition, health-related quality of life (HRQoL) is defined as an individual’s subjective perception of the impact of health status on physical (e.g., illness, pain, fatigue), psychological (e.g., emotional well-being, self-esteem), and social functioning (e.g., friends, family, school), including the ability to perform appropriate daily life activities according to the age of the individual [7]. Therefore, self-esteem reflects the attitude towards oneself and may contribute to better or worse mental health and well-being [8,9]. Furthermore, assessment of HRQoL in children is important to identify subgroups with poor health status, which can help guide effective intervention strategies to improve the health of children [3]. Therefore, a good HRQoL is necessary for the adaptation and healthy growth of children into healthy adolescents and adults in the future [10].

Healthy lifestyle behaviors have been associated with a better quality of life [11]. In this sense, several studies have analyzed the relationship between sedentary time, PA, and HRQoL, but they are mainly focused on adolescents [3,12] or both children and adolescents [13]. Therefore, there is a shortage of studies that only analyze children. Specifically, the review carried out by Wu et al. [13] included a total of 31 studies and only six of them were focused on children. Regarding the Spanish population, to our knowledge, there are only four studies in which PA and HRQoL [14,15,16] or self-reported health status [17] were positively associated, but they focused on adolescents aged 11 to 18 years old.

Moreover, some studies have analyzed the relationship between sedentary behavior, PA, and HRQoL dimensions, specifically with the domains of physical, psychological, and social function factors. In general, the studies showed a positive relationship between sedentary behaviors and higher PA with poor physical, social [18,19], and psychological domains [18,19,20], except one study that reported no association with any HRQoL domain [21]. In addition, only one study analyzed light PA [20], despite it being the predominant level of intensity during the day [22], as well as it contributing to children’s health as demonstrated by improvements in metabolic biomarkers [23].

The HRQoL varies in children by sex at different ages [24], and males tend to be more active than females [6]. In this sense, few studies have analyzed the association between physical activity and HRQoL separately by sex. A study of Australian children aged 8–10 showed that greater participation in team sports was associated with better quality of life in each of the dimensions (physical and psychological health and social, emotional, and school functioning). However, this relationship was stronger in females than males [25]. Another study of Japanese children observed a positive relationship between healthy habits (lower screen time and higher PA) and better quality of life in physical fitness, social activities, and general health in both males and females. Nonetheless, this relationship was stronger in males, while males had greater social support [26].

Consequently, as a result of the importance of the relationship between these factors on children’s health [11], more studies are needed to analyze the associations between sedentary time and different PA and HRQoL [3]. This study also offers new results separated by sex [6], which may show different perceptions of quality of life. Furthermore, the use of objective assessments of sedentary time and PA are vital to understand these associations. Therefore, the aims of this study were: (1) to analyze the sedentary time, objectively measured PA levels (light, moderate, vigorous, and moderate-to-vigorous), and HRQoL dimensions (physical well-being, emotional well- being, self-esteem, family, friends, school, and total score) in children; and (2) to examine the association between sedentary time, PA levels, and HRQoL in children separately by sex.

## 2. Materials and Methods

### 2.1. Participants

The data presented in this manuscript were obtained as part of the Previene Project (Promoting Healthy Lifestyles in the School Environment) [27]. This project has three intervention programs aimed at increasing PA time (active commuting to school, Physical Education lessons, and school recess) and a fourth program aimed at improving sleep hygiene [27]. To participate in this study, the schools had to present at least two grade 3 classes with an average class size of at least 25 children. The baseline data of this study were collected from 15 schools with 3rd grade classes in Granada (Spain) selected by non-randomized sample, in two different stages: firstly, between January–March 2017, and secondly, the same period in 2018. Initially, 717 children from 15 schools were invited to participate. Thereafter, 258 children were excluded from data analysis as 144 did not give informed consent, 17 did not attend the evaluation day, 70 did not wear the accelerometer for 7 days, and 27 did not deliver the log. The final sample consisted of 459 children (8.4 ± 0.4 years old, 50.54% males).

After the schools approved their participation in the study, the research team conducted an initial meeting with the teachers to explain the evaluation process that would be carried out. The families received an invitation to a meeting to receive information about the demands of this project, evaluation dates, evaluation tests, the use and care of the accelerometers, and to encourage their participation in the study. Parents signed an informed consent form for their children to be included in the study. The study protocol was approved by the University of Granada Human Research Ethics Commit- tee (Reference: 57/CEIH/2015).

### 2.2. Instruments

All the instruments that are detailed below were used by the research team at schools during class time to assess the different variables.

#### 2.2.1. Anthropometry

The children’s body mass and stature were assessed wearing shorts and a short sleeve shirt, with bare feet. Body mass was measured with a 0.1 kg approximation using a Seca 876 weighing system (Seca, Ltd., Hamburg, Germany). Stature was measured in the Frankfort plane, with an approximation of 0.1 cm using a Seca 213 stadiometer (Seca, Ltd., Hamburg, Germany). Stature and body mass were measured twice, taking the average of both measurements. Body mass index (BMI) was calculated as body mass in kilograms divided by stature in meters squared. To determine the body mass status of children, we used the age and sex BMI cut-off points proposed by the International Obesity Task Force [28].

#### 2.2.2. Sedentary Time and Physical Activity Levels

The sedentary time and PA were measured using a tri-axial accelerometer (Actigraph wGT3X-BT, Pensacola, FL, USA), which is considered a valid and reliable tool to objectively measure PA in children [29]. The research team attached the accelerometers to the participants’ non-dominant wrist and instructed them on how to take care of the accelerometers. The accelerometers were worn by the children for 7 consecutive days, 24 h/day [30]. In addition, the parents were also asked to complete a log to determine the time that their children were out of bed, bathing/showering or involved in other water activities. All these situations were considered as non-wearing time and excluded from the analyses. The minimum amount of time that was considered acceptable for inclusion in the sample was at least 5 days with at least 10 h per day, including 1 weekend day.

To estimate the children’s PA, the Chandler algorithm was used [31]. The data were processed at a sampling rate of 90 Hz, set to record in 5-s epochs using Actilife software (version 6.8.1). The cut-points to determine the type of intensity were: sedentary time (˂305 counts per 5 s), light PA (306–817 counts per 5 s), moderate PA (818–1968 counts per 5 s), and vigorous PA (˃1969 counts per 5 s).

#### 2.2.3. Health-Related Quality of Life

The children’s HRQoL was evaluated using Revidierter KINDer Lebensqualitätsfragebogen (KINDL-R) validated for Spanish children aged 4 to 16 [32]. This questionnaire consists of 24 items associated with six dimensions of HRQoL: physical well-being (e.g., illness, pain, fatigue), emotional well-being (e.g., boredom, loneliness, scared), self-esteem (e.g., pride, feeling on top of the world), family (e.g., relationship with parents, conflicts at home), friends (e.g., getting along with others or feeling different), everyday functioning in school (e.g., enjoying classes, worrying about the future), and disease (e.g., illness uncertainty, parent overprotection, absence from school) [33]. The total scores from the children’s questionnaires were converted to a scale of 0 to 100, where higher scores indicated better HRQoL. The reliability of this questionnaire in each dimension was measured by α Cronbach coefficient: physical well-being (0.48), emotional well-being (0.54), self-esteem (0.76), family (0.57), friends (0.54), school (0.50), and total score (0.81) [32].

### 2.3. Data Analyses

Descriptive statistics (mean, standard deviation) were calculated for all measured variables. To Kolmogorov–Smirnov test was used to examine the normality of all variables. To examine the differences by sex, independent *t*-tests were conducted. Interactions between sex and sedentary time, PA levels, and HRQoL dimensions were previously tested. Afterwards, linear regressions were applied to analyze the association between sedentary time, PA levels, and HRQoL dimensions. In this statistical test, BMI and sex were included as covariates. The Spearman’s correlation was used to calculate the strength of the relationship between PA levels and HRQoL dimensions, as follows: very weak (0–0.19), weak (0.20–0.39), moderate (0.40–0.59), strong (0.60–0.79), very strong (0.80–1.0) [34]. The level of statistical significance was set at *p* < 0.05 and the confidence level for intervals was always 95%. All statistical analyses were performed using SPSS (version 23.0; SPSS, IBM, Chicago, IL, USA).

## 3. Results

Table 1 presents the descriptive data by sex. Males spent 7.55 min less doing light PA, but did 5.81 min more of vigorous PA and 6.31 min more of moderate-to-vigorous physical activity (MVPA) than females (all, *p* < 0.05). No differences were found in the HRQoL dimensions in this study between males and females.

Table 2 shows the associations between sedentary time, PA levels, and HRQoL dimensions. Sedentary time was negatively associated with self-esteem and total score (all *p* < 0.05). Nonetheless, all PA levels (light, moderate, vigorous, and moderate-to-vigorous) were positively related to self-esteem and total score (all *p* < 0.05).

Table 3 shows the associations between sedentary time, PA levels, and HRQoL dimensions by sex. Sedentary time was negatively associated with more HRQoL dimensions (physical well-being, self-esteem, friends, and total score) in males than females (self-esteem and family) (all *p* < 0.05). Regarding PA levels, light PA was positively associated with more HRQoL dimensions in females (self-esteem, family, and total score) (all *p* < 0.05), while PA levels (moderate, vigorous, and moderate-to-vigorous) were only associated with HRQoL dimensions (physical well-being, self-esteem, friends, and total score) in males (all *p* < 0.05).

## 4. Discussion

The main findings of this study were: (i) females performed more light PA than males, while males engaged in more vigorous PA and MVPA; (ii) sedentary time was negatively associated with self-esteem and total score, while all levels of PA were positively related to self-esteem and total score; (iii) by sex, sedentary time was negatively associated with more HRQoL dimensions in males than females. Regarding PA levels, light PA was positively related to HRQoL dimensions in females, while in males, higher PA intensities were positively associated with more HRQoL dimensions.

We observed that males spent more time engaged in moderate and vigorous PA than females. This result is consistent with the scientific literature. A study carried out among children aged 9–11 from 12 countries showed that males spent 7 min more engaged in vigorous PA and 18 min more engaged in MVPA than females [35]. Similarly, other studies in children reported higher MVPA in males, showing significant differences by sex of between 11 min [36] and 8 min [37]. These differences by sex may be influenced by several factors besides the participation of females in sports activities, such as the biological changes experienced during puberty, lack of motivation in activities that require greater motor commitment, and lower parental support for the practice of sports [38]. Therefore, it is necessary to develop coeducational interventions to promote PA through activities and sports that favor equality between males and females with the aim of reducing these differences. On the other hand, we also observed higher MVPA in our study compared to other studies in children in which PA was objectively measured with an accelerometer placed on the waist or hip. These differences in MVPA could be related to the part of the body to which the accelerometers were attached. In this sense, a study by Kumahara et al. [39] analyzed PA in children using accelerometers at the wrist and waist, showing a greater number of accelerations in the wrist than in the waist.

Sedentary time was negatively associated with self-esteem and total score, while PA was positively associated with self-esteem and total score. Thus, PA, self-esteem, and total score presented a moderate correlation between them (Table S1). Previous studies in children also presented a positive relationship between low sedentary behaviors and HRQoL [15,26,40,41,42]. Higher sedentary time, such as screen time, is related to greater stress or anxiety and influences psychological ill-being [43] contributing to lower self-esteem [44]. In addition, a systematic review carried out by Marker et al. [3] in children and adolescents observed a positive relationship between PA and HRQoL. Similarly, another systematic review focused on children and adolescents also reported a positive association between PA and self-esteem [45]. This relationship was explained in other studies that indicated that participation in sports activities improves the development of motor skills, which contributes to an improvement in autonomy, thereby improving self-esteem [46]. In addition, it can contribute to an improvement in social relationships through the interaction between the participants in the sport, as well as an increase in enjoyment which contributes to improved self-esteem [47]. Physical activity is presented as a good tool to increase quality of life due to the psychosocial benefits and the possibilities for social interaction and independence for children [48]. Nonetheless, among the different mental health benefits that physical activity can provide, the strongest effects seem to be related to self-esteem [49]. However, this is a cross-sectional study so we should assume correlation rather than causality, as adolescents with higher self-esteem may engage in activities with higher intensity because they feel capable of this. Therefore, on the basis of the outcomes shown in our study, as well as the confirmation of these results with different systematic reviews, it is necessary to carry out intervention programs aimed at reducing sedentary time and increasing daily PA in children.

In relation to sex, sedentary time was negatively associated with more HRQoL dimensions in males than females. To our knowledge, there are no previous studies in children evaluating sedentary time and HRQoL dimensions separated by sex. Nonetheless, these differences could be due to the fact that females are usually more sedentary while males are more physically active [50]. The fact that PA is positively related to better HRQoL [3] and males are less sedentary than females [50] could partly explain the findings of our study. Regarding the relationship between PA and HRQoL dimensions, we observed that light PA was positively related to HRQoL dimensions in females, while in males, higher PA intensities were positively associated with more HRQoL dimensions. In this sense, a systematic review indicated that both light PA and MVPA were positively related with better quality of life; however, these relationships were more consistent and robust for MVPA than light PA [23]. As females tend to be less active than males [6], they are likely to achieve health benefits at lower intensity activity. Therefore, smaller amounts of PA in females could improve their perception of quality of life more than in males. In addition, this could be related to changes in leisure activities, where females preferred to do other types of activities that require a lower motor skill levels, for instance light-intensity walking [38]. In relation to the aforementioned, a study in adolescents showed low self-esteem in females when the proposed sports activities did not encourage their participation, as they were sometimes humiliated by their classmates [51]. This also occurred in activities that focus on competition [41]. This situation can contribute to females not participating in team sports activities because they feel they will not be successful [52]. In relation to males, greater participation in sports activities contributes to promoting social relationships [53] as well as an improvement in physical well-being produced by the benefits of PA on health [54]. However, it is important to analyze these associations with larger samples to verify the differences in the associations between males and females. On the basis of the results obtained in our study, we consider it necessary to carry out programs in the school environment with the aim of promoting PA among children due to its positive relationship with HRQoL. In addition, it is especially important to increase PA in females as they presented a lower PA time. It is particularly recommended because increasing PA is related to higher self-esteem. Specifically, there is a strong association between school-based interventions for PA with self-esteem and self-concept [55]. In addition, we believe that future school programs should take sex differences into account in order to achieve health-related benefits of PA in males and females, as it has been proved that current intervention programs are not equally effective for males and females [56]. Therefore, future studies should promote PA by including activities that alternate between light and moderate-to-vigorous intensity.

This study has various strengths such as the use of an objective tool to measure PA (accelerometer), which offers more accurate data compared to other self-reported tools. Moreover, as a result of the scarcity of studies of this type in the Spanish population, we offer new outcomes to better understand the relationship between sedentary time, PA levels, and HRQoL. Nevertheless, this study also has certain limitations. For example, the schools were recruited by non-randomized sample. Furthermore, although the PA was evaluated with accelerometers, its placement on the wrist showed higher amounts of movements compared to other parts of body such as the waist or hip [57], thus the results should be considered with this in mind. In addition, raw data may be used in future studies. Despite this, it is worth noting that a recent review proposed this accelerometer position as a suitable area to measure PA in children [30].

## 5. Conclusions

This study presents updated results of sedentary time, PA, and HRQoL in Spanish children. Our findings indicate that females spent more time engaged in light PA, while males spent more engaged in vigorous PA and MVPA. Both sedentary time and PA levels were associated with self-esteem and total score. However, sedentary time was negatively associated with more HRQoL dimensions in males than females, while light PA had a greater impact on females’ HRQoL than on that of males. It is important to increase PA levels in males and females due to its relation with HRQoL. In addition, intervention programs should develop activities with different intensities in order to be effective for both males and females.

## Figures and Tables

**Table 1:** Descriptive data of participants’ physical activity (PA) levels and health-related quality of life (HRQoL) dimensions divided by sex.

	Total Mean ± SD	Males’ Mean ± SD	Females’ Mean ± SD	*p*-Value
*n*	459	232	227	
Age (years)	8.45 ± 0.35	8.48 ± 0.36	8.43 ± 0.33	0.153
Weight (Kg)	31.15 ± 6.86	31.46 ± 6.95	30.84 ± 6.77	0.337
Height (m)	1.32 ± 0.05	1.32 ± 0.05	1.31 ± 0.05	**0.008**
BMI (Kg/m^2^)	17.73 ± 2.99	17.70 ± 2.88	17.76 ± 3.10	0.822
**PA**				
Sedentary time (min/day)	502.73 ± 54.88	504.42 ± 54.29	500.99 ± 55.54	0.413
Light PA (min/day)	233.01 ± 30.86	229.27 ± 29.89	236.82 ± 31.44	**0.009**
Moderate PA (min/day)	94.14 ± 23.71	94.38 ± 22.90	93.89 ± 24.56	0.824
Vigorous PA (min/day)	11.55 ± 6.73	14.42 ± 7.27	8.61 ± 4.54	**<0.001**
MVPA (min/day)	105.69 ± 27.90	108.81 ± 27.87	102.50 ± 27.62	**0.015**
**HRQoL**				
Physical well-being	77.15 ± 17.84	78.36 ± 16.27	75.90 ± 19.27	0.140
Emotional well-being	84.95 ± 16.35	84.83 ± 16.07	85.07 ± 16.67	0.873
Self-esteem	77.55 ± 23.14	77.07 ± 23.62	78.05 ± 22.69	0.650
Family	83.68 ± 15.71	83.24 ± 15.06	84.14 ± 16.38	0.541
Friends	82.92 ± 15.69	82.46 ± 14.86	83.39 ± 16.52	0.524
School	68.04 ± 18.33	66.56 ± 16.76	69.54 ± 19.72	0.082
Total score	77.31 ± 10.29	77.20 ± 9.60	77.42 ± 10.97	0.821

SD: standard deviation, BMI: body mass index; PA: physical activity; MVPA: moderate-to-vigorous physical activity; HRQoL: health-related quality of life. Significant values are highlighted in bold.

**Table 2:** Association between sedentary time, PA levels, and HRQoL dimensions.

	Sedentary Time	Light PA	Moderate PA	Vigorous PA	MVPA
	β	β	β	β	β
	(CI)	(CI)	(CI)	(CI)	(CI)
Physical well-being	−0.23	0.43	0.3	2.05	0.31
	(−0.50, 0.04)	(−0.05, 0.91)	(−0.29, 0.88)	(−0.25, 4.35)	(−0.19, 0.81)
Emotional well-being	−0.10	0.12	0.31	1.87	0.32
	(−0.35, 0.15)	(−0.43, 0.46)	(−0.22, 0.85)	(−0.24, 3.98)	(−0.14, 0.77)
Self-esteem	−0.55	0.74	1.15	4.33	1.04
	**(−0.91, −0.20)**	**(0.12, 1.37)**	**(0.39, 1.90)**	**(1.33, 7.33)**	**(0.39, 1.68)**
Family	−0.12	0.22	0.16	1.01	0.16
	(−0.36, 0.12)	(−0.20, 0.65)	(−0.36, 0.70)	(−1.02, 3.05)	(−0.28, 0.60)
Friends	−0.23	0.32	0.48	1.31	0.41
	(−0.47, 0.01)	(−0.11, 0.74)	(−0.03, 0.99)	(−0.72, 3.34)	(−0.02, 0.85)
School	−0.15	0.28	0.25	0.42	0.20
	(−0.43, 0.13)	(−0.21, 0.78)	(−0.35, 0.84)	(−1.94, 2.76)	(−0.31, 0.71)
Total score	−0.20	0.30	0.36	1.40	0.33
	**(−0.35, −0.04)**	**(0.02, 0.58)**	**(0.03, 0.70)**	**(0.08, 2.73)**	**(0.05, 0.62)**

CI: Confidence interval; PA: physical activity; MVPA moderate-to-vigorous physical activity. Significant values are highlighted in bold.

**Table 3:** Association between sedentary time, PA levels, and HRQoL dimensions by sex.

	Sedentary Time		Light PA		Moderate PA		Vigorous PA		MVPA	
	Males	Females	Males	Females	Males	Females	Males	Females	Males	Females
	β	β	β	β	β	β	β	β	β	β
	(CI)	(CI)	(CI)	(CI)	(CI)	(CI)	(CI)	(CI)	(CI)	(CI)
Physical well-being	−0.46	−0.02	0.54	0.32	1.01	−0.35	3.02	−0.42	0.88	−0.29
	**(−0.82, −0.10)**	(−0.43, 0.39)	(−0.09, 1.17)	(−0.41, 1.06)	**(0.25, 1.78)**	(−1.22, 0.51)	**(0.55, 5.50)**	(−5.17, 4.33)	**(0.26, 1.51)**	(−1.06, 0.48)
Emotional well-being	−0.06	−0.14	−0.24	0.25	0.41	0.26	2.10	1.45	0.41	0.22
	(−0.41, 0.30)	(−0.49, 0.22)	(−0.87, 0.39)	(−0.39, 0.88)	(−0.35, 1.18)	(−0.52, 0.97)	(−0.35, 4.56)	(−2.63, 5.53)	(−0.21, 1.04)	(−0.45, 0.88)
Self-esteem	−0.56	−0.54	0.24	1.25	1.63	0.71	6.24	−0.78	1.50	0.54
	**(−1.08, −0.04)**	**(−1.03, −0.06)**	(−0.68, 1.15)	**(0.38, 2.11)**	**(0.52, 2.73)**	(−0.33, 1.75)	**(2.71, 9.77)**	(−6.49, 4.89)	**(0.60, 1.41)**	(−0.39, 1.46)
Family	0.21	−0.43	−0.37	0.78	−0.48	0.73	0.65	1.81	−0.28	0.62
	(−0.12, 0.55)	**(−0.77, −0.08)**	(−0.96, 0.21)	**(0.18, 1.41)**	(−1.20, 0.23)	(−0.01, 1.46)	(−1.66, 2.96)	(−2.21, 5.84)	(−0.87, 0.31)	(−0.03, 1.27)
Friends	−0.5	0.03	0.61	0.02	1.12	−0.09	2.71	−2.02	0.93	−0.12
	**(−0.82, −0.18)**	(−032, 0.38)	**(0.04, 1.19)**	(−0.61, 0.65)	**(0.42, 1.81)**	(−0.84, 0.66)	**(0.45, 4.96)**	(−6.07, 2.04)	**(0.36, 1.50)**	(−0.78, 0.54)
School	−0.33	0.01	0.34	0.23	0.79	−2.24	2.13	−0.41	0.67	−0.29
	(−0.70, 0.04)	(−0.41, 0.43)	(−0.31, 0.99)	(−0.52, 0.98)	(−0.01, 1.58)	(−1.12, 0.65)	(−0.42, 4.68)	(−0.83, 0.80)	**(0.02, 1.32)**	(−1.08, 0.50)
Total score	−0.23	−0.16	0.18	0.41	0.60	0.15	2.21	−0.69	0.55	0.09
	**(−0.45, −0.02)**	(−0.39, 0.07)	(−0.20, 0.55)	**(0.01, 0.83)**	**(0.15, 1.05)**	(−0.35, 0.64)	**(0.77, 3.66)**	(−3.38, 2.01)	**(0.18, 0.92)**	(−0.34, 0.54)

CI: Confidence interval; PA: physical activity; MVPA moderate-to-vigorous physical activity. Significant values are highlighted in bold.

## Supplementary Materials

The following are available online at https://www.mdpi.com/1660-4601/18/5/2702/s1, Table S1: Correlation between PA levels and HRQoL dimensions.
