# Supplementary material for: Relationship between Sedentary Time, Physical Activity, and Health-Related Quality of Life in Spanish Children
Source: Int J Environ Res Public Health. 2021 Mar 8;18(5):2702. doi: 10.3390/ijerph18052702 (PMC7967425; doi:10.3390/ijerph18052702)
Supplement: Supplementary file 1 [file ijerph-18-02702-s001.pdf]

Additional file presents the correlation analysis between the HRQoL dimensions and PA levels. Self-esteem presented a low correlation with PA (light, moderate, vigorous, and moderate-to-vigorous) (all, r <0.2). The total score obtained a low correlation with PA (moderate, and moderate-to-vigorous) (all, r <0.2).

Table S1. Correlation between PA levels and HRQoL dimensions.

	**Light PA**		**Moderate PA**		**Vigorous PA**		**MVPA**	
	**R**	***P***	**R**	***P***	**R**	***P***	**R**	***P***
Physical well-being	0.08	0.09	0.07	0.15	0.09	0.05	0.08	0.09
Emotional well-being	−0.01	0.75	0.04	0.35	0.07	0.14	0.06	0.21
Self-esteem	0.11	**0.02**	0.16	**0.00**	0.10	**0.04**	0.16	**0.00**
Family	0.06	0.21	0.07	0.14	0.03	0.58	0.07	0.15
Friends	0.08	0.08	0.07	0.11	0.03	0.51	0.08	0.10
School	0.05	0.24	0.03	0.54	−0.03	0.48	0.02	0.66
Total score	0.09	0.06	0.11	**0.02**	0.08	0.09	0.11	**0.01**

PA: physical activity; MVPA: moderate-to-vigorous physical activity. Significant values are highlighted in bold
